# Supplementary material for: Testing the Development of a Diet-Based Bisphenol a Score to Facilitate Studies on Child Neurodevelopment: A Pilot Project
Source: Int J Environ Res Public Health. 2025 Jul 25;22(8):1174. doi: 10.3390/ijerph22081174 (PMC12386539; doi:10.3390/ijerph22081174)
Supplement: Supplementary file 1 [file ijerph-22-01174-s001.zip › ijerph-3674496-supplementary.pdf]

Supplemental Material

Title: Testing the development of a diet-based Bisphenol A score to facilitate studies on child neurodevelopment: A pilot project

Authors: Marisa A. Patti, Apollo Kivumbi, Juliette Rando, Ashley Song, Lisa A. Croen, Rebecca J. Schmidt, Heather Volk, Kristen Lyall

Contents **Page #**

Table S1. Components of BPA dietary burden score…………………………….... 2
Table S2. Univariate statistics of repeated maternal urinary concentrations of BPA.. 4
Table S3. Associations between BPA dietary burden scores and average gestational urinary $\log_{10}$ BPA concentrations and SRS T-scores, with additional adjustment for health-related behaviors……………………………………………………….... 5
Figure S1. Flow chart of participant selection to final sample size………………… 6
Figure S2. Directed acyclic graph referenced when selecting covariates for adjusted analyses……………………………………………………………….. 7
Figure S3. Kernel density plot of children’s SRS T-sores………………………... 8

Table S1. Components of BPA dietary burden score

Item: Question from adapted diet history questionnaire and food preparation forms	Assigned Weight[a]	ICC value
How often were the vegetables you ate canned?<br>• Always or almost always (at least 90% of the time)<br>• Somewhat frequently (50-89% of the time)<br>• Once in awhile (10-49% of the time)<br>• Rarely or never (less than 10% of the time)<br>• Don't know	1	0.77 (0.65, 0.85)
How often were the fruits you ate canned?<br>• Always or almost always (at least 90% of the time)<br>• Somewhat frequently (50-89% of the time)<br>• Once in awhile (10-49% of the time)<br>• Rarely or never (less than 10% of the time)<br>Don't know	1	0.65 (0.49, 0.77)
Since the beginning of your pregnancy, how often did you eat prepackaged foods or meals in a plastic dish or container?<br>• Never<br>• Less than once a month<br>• 1-3 times a month<br>• Once a week<br>• 2-3 times a week<br>• 4-6 times a week<br>• Once a day<br>• More than once a day<br>• Refused<br>• Don't know	0.25	0.34 (0.18, 0.48)
Since the beginning of your pregnancy, how often did you microwave food in a plastic container for yourself to eat?<br>• Never<br>• Less than once a month<br>• 1-3 times a month<br>• Once a week<br>• Once every 2 weeks<br>• Once a week<br>• Every other day<br>• Once a day<br>• Several times a day<br>• Refused<br>• Don't know	0.25	0.72 (0.63, 0.79)
Since the beginning of your pregnancy, did you use plastic containers to store food in your household?<br>• Yes<br>• No<br>• Refused<br>• Don't know	0.25	0.61 (0.48, 0.70)
Since the beginning of your pregnancy, did you use plastic wrap to store food in your household?<br>• Yes<br>• No<br>• Refused	0.25	0.58 (0.46, 0.69)

• Don't know		
Since the beginning of your pregnancy, how many times per week do you eat fast food (including Chinese take-out)<br>• Never<br>• Less than once per week<br>• 1-3 times per week<br>• 4-6 times per week<br>• 7-9 times per week<br>• 10-12 times per week<br>• 13-14 times per week<br>• 15 or more times per week<br>• Refused<br>• Don't know	0.25	0.50 (0.36, 0.62)

ICC: Intra class correlation coefficient
[a] Weights based off prior work to develop assessments of dietary based BPA contamination [27, 28]

Table S2. Univariate statistics of repeated maternal urinary concentrations of BPA (ng/mL)

N	226
Percent > LOD	88
Minimum	0.14
Maximum	39.10
5th Percentile	0.30
25th Percentile	0.60
Median	1.10
75th Percentile	2.10
95th Percentile	4.73
Intraclass correlation coefficient (ICC) (95% CI)	0.48 (0.33, 0.61)

Table S3. Associations between BPA dietary burden scores and average gestational urinary log$_{-10}$ BPA concentrations and SRS T-scores, with additional adjustment for health-related behaviors

	Original Analyses		Sensitivity Analyses			
	Unadjusted<br>β (95% CI)	Adjusted [1]<br>β (95% CI)	Non-smokers[2]<br>β (95% CI)	Plus Brest feeding[3]<br>β (95% CI)	Prenatal vitamins[4]<br>β (95% CI)	Plus HEI[5]<br>β (95% CI)
Association between BPA dietary burden scores and SRS T-scores						
	0.01<br>(-0.01, 0.04)	0.00<br>(-0.03, 0.03)	0.00<br>(-0.04, 0.03)	0.00<br>(-0.03, 0.03)	0.00<br>(-0.04, 0.03)	0.00<br>(-0.03, 0.04)
Association between urinary BPA concentrations and SRS T-scores						
	0.00<br>(0.00, 0.01)	0.00<br>(-0.01, 0.01)	0.00<br>(-0.01, 0.00)	0.00<br>(-0.01, 0.01)	0.00<br>(-0.04, 0.01)	0.00<br>(0.00, 0.01)

BPA: Bisphenol A, ICE: Index of Concertation at the Extremes, HEI: Healthy Eating Index

[1] Adjusted for maternal age (continuous), education (high school or less v. some college v. completed college), pre pregnancy BMI (continuous), and additionally maternal race & ethnicity (non-white v. Hispanic and or Black or other race), gestational cotinine concentration (continuous, proxy for tobacco smoke exposure), and annual household income (<\$50k, \$50-<\$100k, >\$1000k)

[2] Analyses were restricted to only include those who were self-reported non-smokers during pregnancy (n=91, excluding n=20 with missing self-reported smoking information and n=5 self-reported smokers or self-reported household smoke exposure)

[3] Additionally adjusted for breast feeding status (yes v. no), sample was restricted to those with available data (n=104)

[4] Analyses were restricted to only include those who self-reported took prenatal vitamins during pregnancy (n=110, excluding n=6 self-reported did not take any prenatal vitamins during pregnancy)

[5] Additionally adjusted for the American healthy eating index, where sample was restricted to include those with available data (n=113)

Figure S1. Flow chart of participant selection to final sample size

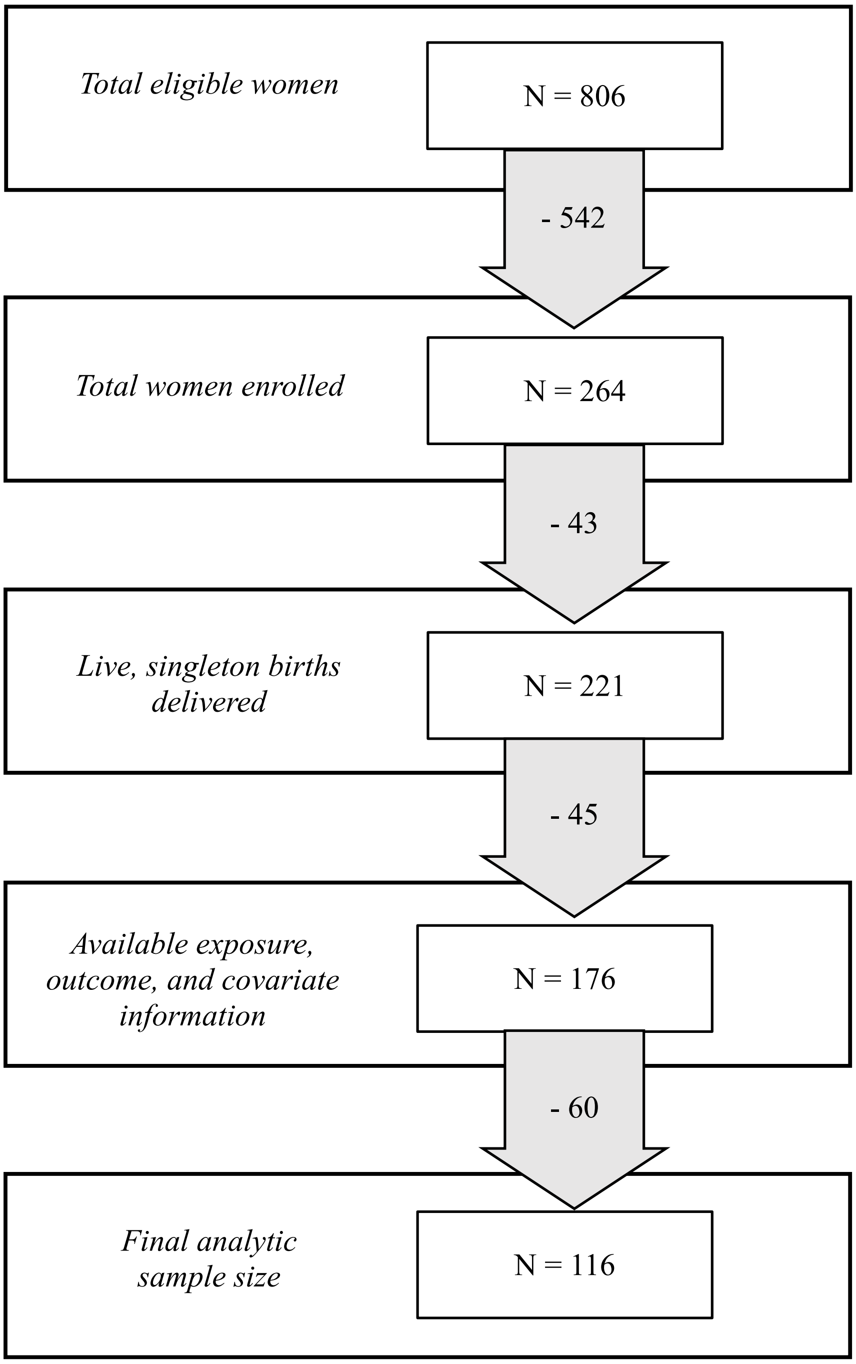

Figure S2. Directed acyclic graph referenced when selecting covariates for adjusted analyses

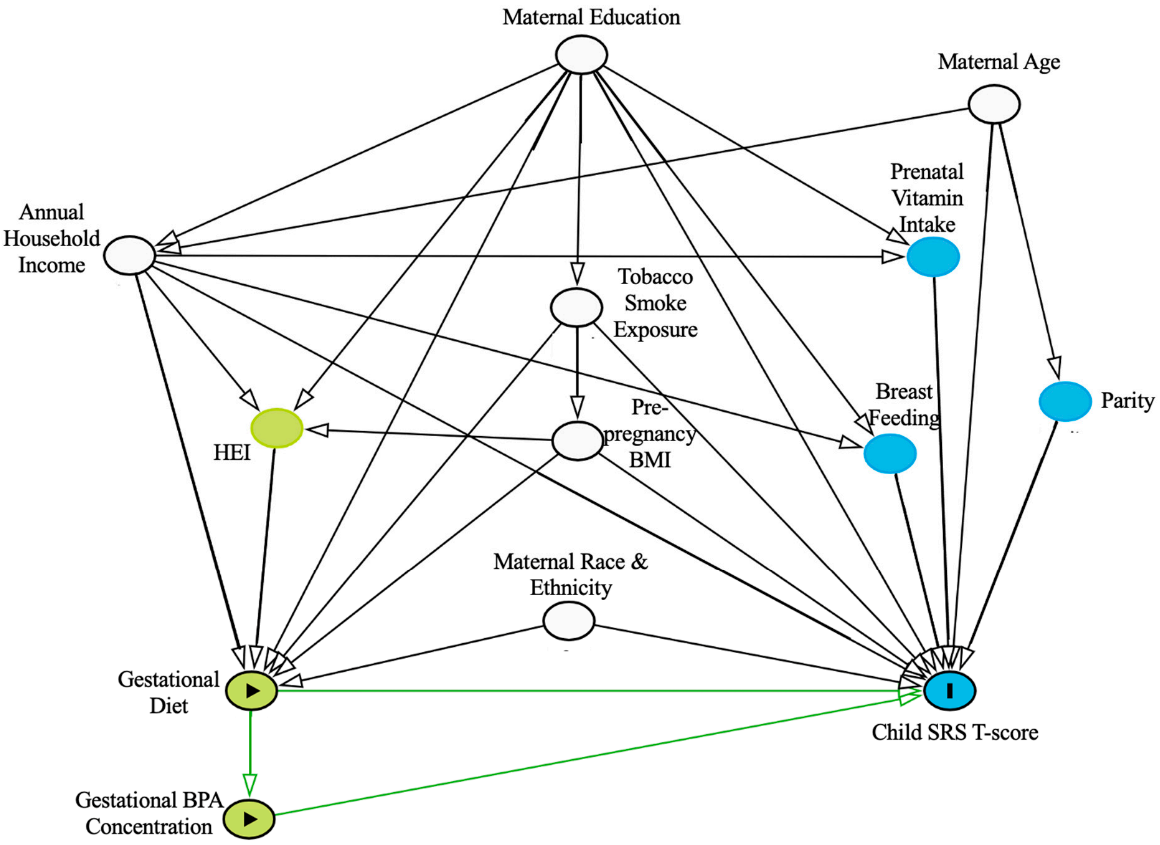

Exposure	Outcome	Ancestor of Exposure	Ancestor of Outcome	Ancestor of Exposure and Outcome	Adjusted variable	Causal Path	Unbiased Path

Figure S3. Kernel density plot of children's SRS T-sores

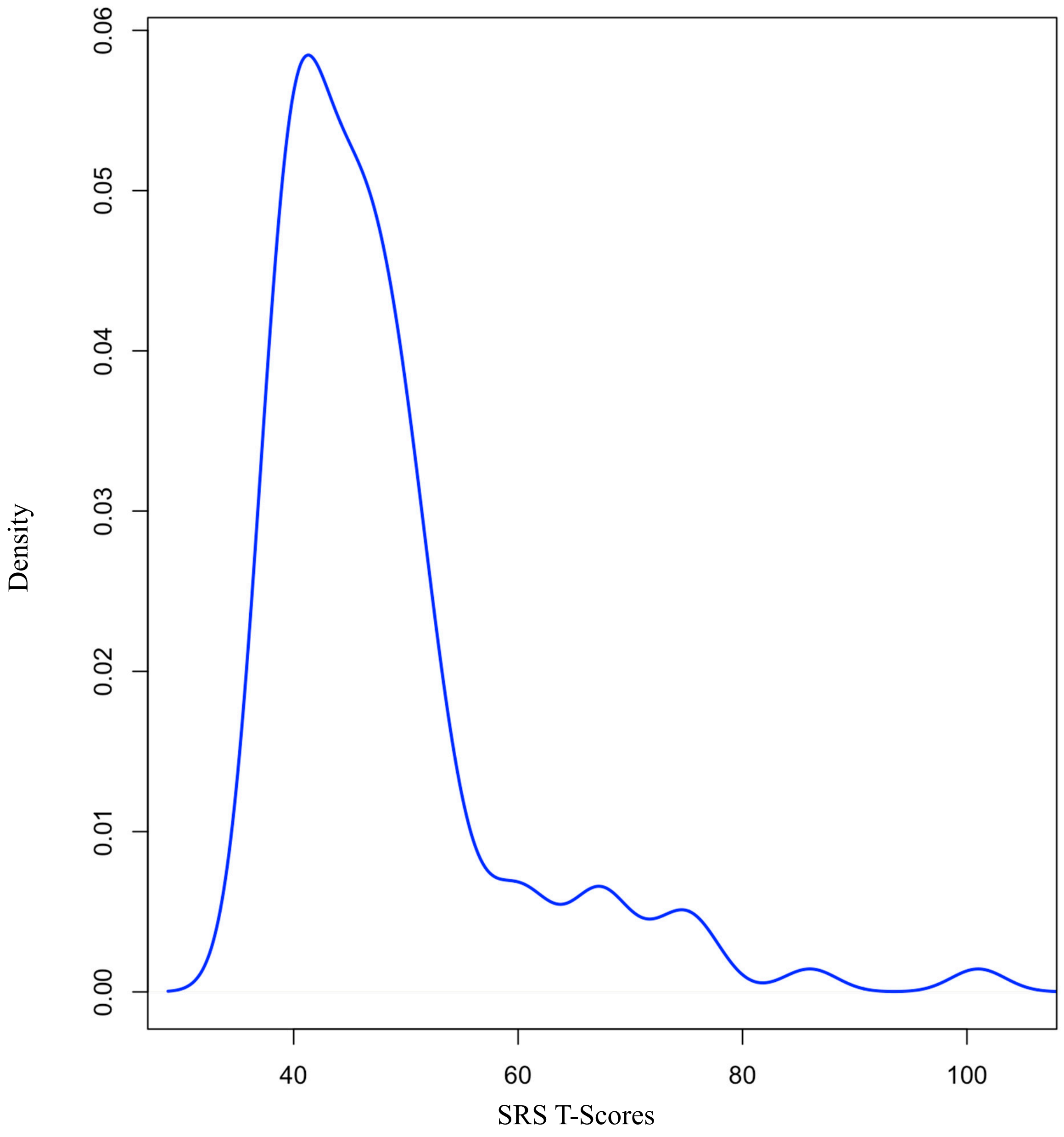

Child SRS T-scores had a mean of 48 and standard deviation of 11. The proportion of children with SRS scores ≥60 was 12% and the proportion of children with SRS scores ≥75 was 4%. SRS T-scores ranging from 60–75 are indicative of clinically significant deficiencies in reciprocal social behavior that may interfere with daily social interactions, while scores greater than 75 are strongly associated with clinical diagnosis of autism.
